# Characteristics and risk factors of fever after total joint arthroplasty: a single-center retrospective study

**DOI:** 10.1186/s12891-022-05940-3

**Published:** 2022-11-12

**Authors:** MingYang Li, ChengYu Lyu, Yuan Fang, ZhenShuai Shao, Kun Liu, Ning Liu

**Affiliations:** 1 Department of Joint Surgery, The Affiliated Hospital of Qingdao University, Qingdao, 266000 Shandong China; 2 Shanghai Maritime University, Shanghai, 200120 China

**Keywords:** Total Knee Arthroplasty, Total Hip Arthroplasty, Postoperative Fever

## Abstract

**Background:**

Postoperative fever (POF) is a common problem after total joint arthroplasty (TJA). The goal of this research is to analyze the characteristics and risk factors of fever following TJA.

**Methods:**

We retrospectively investigated 2482 patients who had primary total knee arthroplasty (TKA) or total hip arthroplasty (THA) surgery at our institution between January 2020 and December 2020. Those patients were divided into TKA group and THA group. The patients' axillary temperatures were measured. POF was defined as a body temperature greater than 38 °C. Then patients in the TKA and THA groups were respectively divided into afebrile group and febrile group based on their body temperatures. Temperature changing characteristics of the patients in the febrile group were analyzed and recorded. According to the number of patients in the febrile group, we randomly selected a corresponding number of patients from the afebrile group at a ratio of 1:2 to establish a control group. Gender, hypertension, diabetes, anesthesia, surgical time, and some laboratory data were analyzed between the febrile group and the afebrile group.

**Results:**

Three percent of TKA patients (*N* = 45) had febrile, and in the febrile group of TKA group, 38% (*N* = 17) had fever and maximum body temperature on postoperative day 2(POD2). Six percent of THA patients (*N* = 46) had fever, and in the febrile group of THA group, 65% (*N* = 30) of the patients had fever and maximum body temperature on POD1. In TKA group, compared with afebrile group, febrile group has higher C-reactive protein (mg/L) (CRP) after surgery. In THA group, compared with the afebrile group, the patients in the febrile group had larger fall in hemoglobin (g/L), and higher C-reactive protein (mg/L) (CRP) after surgery, so there were statistically significant differences between the two groups (*P* < 0.05).

**Conclusion:**

The POF rate of TKA is 3%, and the first fever and maximum body temperature most commonly appear on the POD2. THA has a 6% POF rate, and the first fever and the maximum body temperature most commonly appear on the POD1. In both groups, high C-reactive protein is a risk factor for postoperative fever. In addition, the fall in hemoglobin is also related to postoperative fever in the THA group.

## Background

Total joint arthroplasty (TJA) is an effective treatment of diseases such as end-stage osteoarthritis, avascular necrosis and developmental dysplasia of hip joint [1, 2]. Although the success rate of this surgery has reached 90% or above, there are still many problems bothering us, such as fever. Currently it is believed that postoperative fever is a normal physiological response to total joint arthroplasty [3]. However, fever is usually associated with infection and is one of the early symptoms of periprosthetic joint infection (PJI) which is a devastating consequence [4, 5]. In order to avoid it, doctors will conduct a series of examinations on patients with fever, such as blood and urine routines, CRP, erythrocyte sedimentation rate (ESR), procalcitonin (PCT), chest CT, etc. But there is plenty of evidence suggesting that extensive examination of infectious etiology may be unnecessary, because it will increase medical expenses, prolong hospital stays and waste medical resources [6]. Therefore, once the risk factors of fever after TJA found out, we can effectively lower the occurrence rate of fever and diminish the time and expenses on hospitalization. Objective of this study are as follows: 1. To look into the characteristics of fever after TJA; 2. To look into the risk factors of fever after TJA.

## Method

### Study setting

This study retrospectively investigated 2482 patients who had primary TKA or THA surgery at our institution between January 2020 and December 2020 by any one of six surgeons. 12 patients were excluded because they did not meet the inclusion criteria, leaving the rest patients divided into two groups: the TKA group and the THA group. 796 patients were included in the THA group, and 1674 were included in the TKA group. The demographic characteristics of the patients are shown in Table 1. In the TKA group, 11% (*N* = 189) patients received urinary catheter and 44% (*N* = 743) patients received spinal anesthesia. In the THA group,10% (*N* = 81) patients received urinary catheter and 39% (*N* = 311) patients received spinal anesthesia.

**Table 1:** Demographics of patients

Characteristics	TKA group	THA group
Case number	1674 patients	796 patients
Gender (male/female)	434/1240	428/368
Mean age (years)	67.3 ± 8.7	60.7 ± 7.2
Mean BMI (kg/m²)	26.1 ± 7.3	23.6 ± 5.8
Urinary catheter (yes/no)	189/1485	81/715
Anesthetic type(spinal/General)	743/931	311/485

Four doses of one-gram cefazolin were given to each patient through intravenous drip injection as an antibiotic prophylactic (one dose during operation plus three post-operative doses). Drainage tube was placed in a normal way after operation and removed on POD 1. All patients received deep venous thrombosis(DVT)prophylaxis (compression stockings plus low-molecular-weight heparin or rivaroxaban) for two weeks after operation. The patient began functional exercise on POD1. A specialized nurse measured the patient's axillary temperature with a mercury thermometer and recorded it in the medical records at least four times per day (at 06:00, 10:00, 14:00 and 18:00 h). The patients whose temperatures are abnormal should be measured repeatedly.

Fever was defined as a body temperature greater than 38 °C [7, 8]. Changing characteristics of the patient's body temperature was analyzed. Patients in the TKA and THA groups were respectively divided into afebrile group and febrile group based on their body temperatures. In the febrile group, the daily maximum body temperature of patients during hospitalization (pre-operative day, day of operation (POD 0) and POD 1–4, or until discharge) was collected and recorded from medical records.

**Fig. 1:**
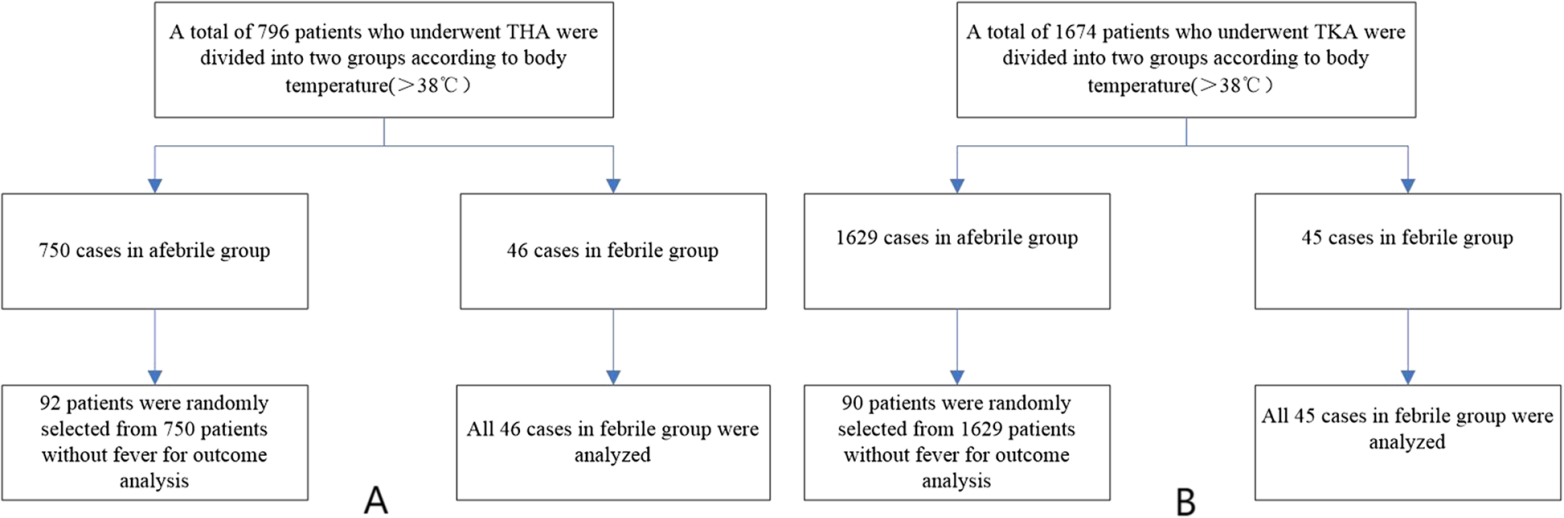
**A**, **B** Flowchart of Patients Grouping

According to the number of patients in the febrile group, we randomly selected a corresponding number of patients from the afebrile group at a ratio of 1:2 to establish a control group (Fig. 1), in which medical records, surgical factors and related test findings of patients in both groups were compared. The patient factors included gender, hypertension and diabetes. Surgical time, postoperative drainage volume, and type of anesthesia (general or spinal) used were all included in surgical factors. Whether a post-operative allogenic blood transfusion was given and whether an auricular catheter was used were also compared factors. Pre-operative and second post-operative day hemoglobin levels, second post-operative day CRP level, and pre-operative albumin level were all tested in the laboratory.

#### Inclusion and exclusion criteria

Inclusion criteria were that patients underwent primary TKA or THA and had no history of open knee or hip surgery(Those patients who had undergone arthroscopic surgery were included in the study too). Patients with cancer or autoimmune diseases were excluded. Infection and inflammatory joint disease were also excluded.

### Statistical analyses

Data analyses and calculations were performed by SPSS Version 25.0 (SPSS Inc., Chicago, IL, USA). Descriptive statistical analyses were conducted on the fever characteristics of patient. The correlations between fever and surgical time, postoperative drainage volume, fall in hemoglobin, second post-operative day CRP level, and preoperative albumin level were analyzed through Students’ T-test. The correlations between fever and gender, hypertension, diabetes, allogenic blood transfusion after operation, use of urinary catheter and anesthetic type were analyzed through the Chi-square Test or Fisher's Exactness Test. A value of *p* < 0.05 was considered statistically significant.

We obtained approval for this retrospective data analysis from the Medical Ethics Committee of the Affiliated Hospital of Qingdao University.

## Results

**Fig. 2:**
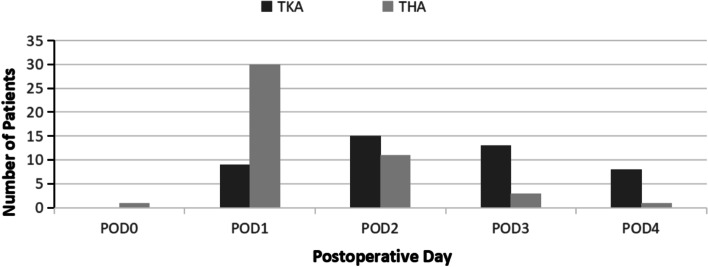
Number of Patients who Developed First Fever after Surgery in both Groups and Corresponding POD

There were 1674 patients in the TKA group, During their hospital stay, Three percent (*N* = 45) developed a fever, in this group, 38% (*N* = 17) on POD2, 33% (*N* = 15) on POD3, 20% (*N* = 9) on POD4, and 9% (*N* = 4) on POD1 (see Fig. 2 and Table 2).

**Table 2:** Number of patients who developed first fever after surgery in both groups and corresponding POD

Group Designation	TKA Group	THA Group	Total No. of Patients
Total no. of patients	1674	796	2470
No. of patients who developed fever	45(3%)	46(6%)	91(4%)
POD 0	0(0%)	1(2%)	1(0%)
POD 1	4(9%)	30(65%)	34(1%)
POD 2	17(38%)	10(22%)	27(1%)
POD 3	15(33%)	2(4%)	17(1%)
POD 4	9(20%)	3(7%)	12(0%)

Seven hundred and ninety-six patients underwent THA, Six percent (*N* = 46) of these patients developed a fever during hospitalization. Of this group, 65% (*N* = 30) patients developed a fever on POD1, 22% (*N* = 10) on POD2, 7% (*N* = 3) on POD4, 4% (*N* = 2) on POD3 and 2% (*N* = 1) on POD1 (See Fig. 2 and Table 2).

**Fig. 3:**
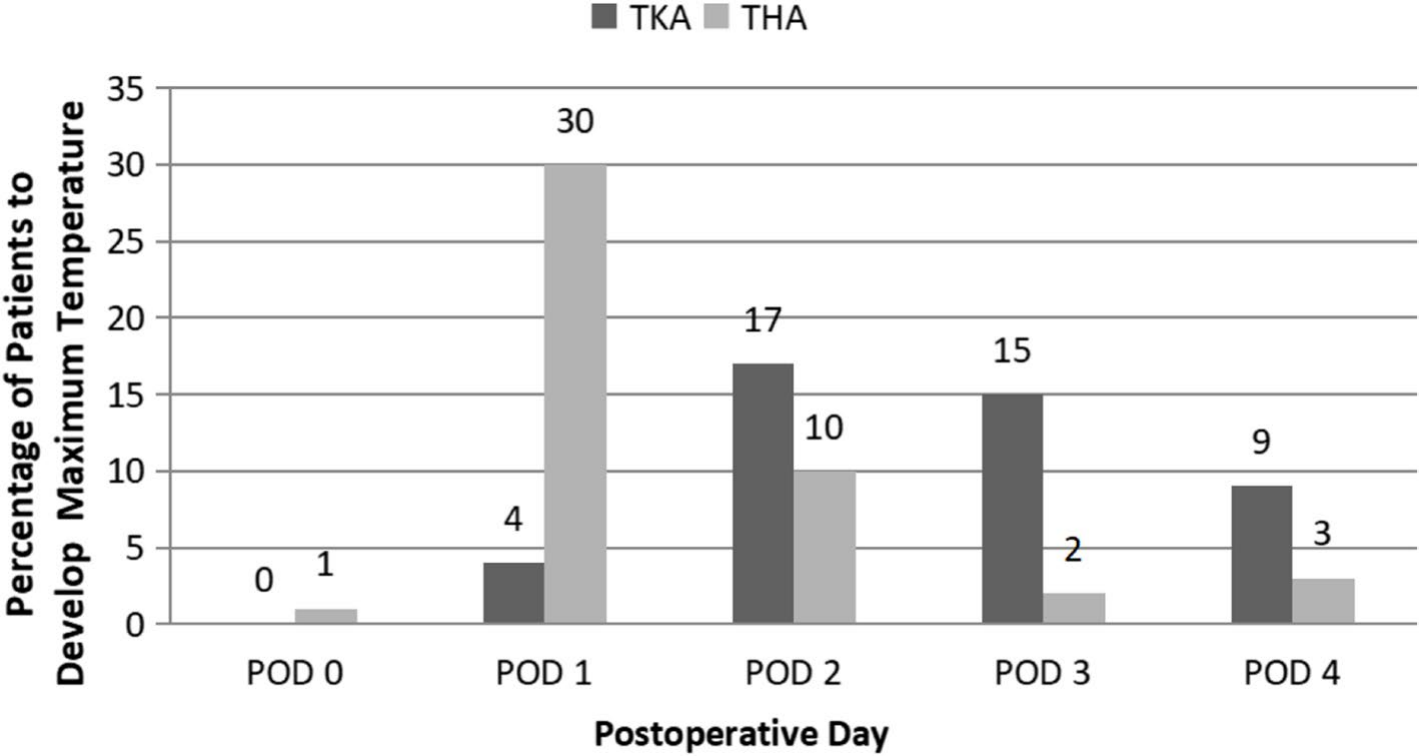
The highest number of patients in TKA groups developed a maximum temperature on POD 2. The highest number of patients in THA groups developed a maximum temperature on POD 1

**Fig. 4:**
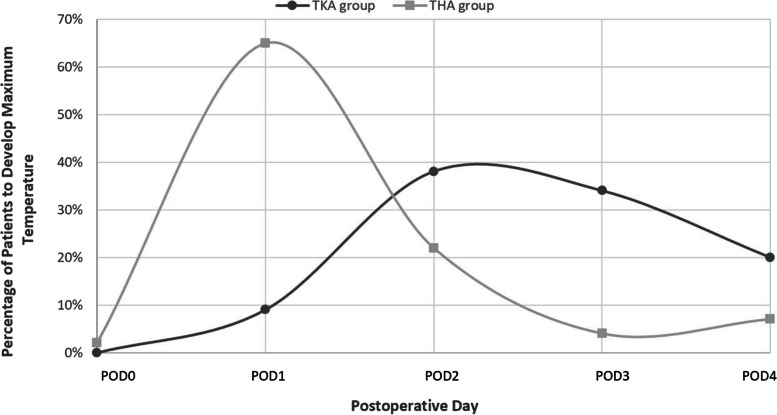
The highest percentage of patients in TKA groups developed a maximum temperature on POD 2 followed by POD 3 The highest percentage of patients in THA groups developed a maximum temperature on POD 1 followed by POD 2

In the TKA group of patients, 9% (*N* = 4) of patients had maximum body temperature on POD1, 38% (*N* = 17) on POD2, 34% (*N* = 15) on POD3, and 20% (*N* = 9) on POD4. No patient developed fever on POD0 (See Figs. 3, 4 and Table 3).

**Table 3:** Number of patients who developed maximum body temperature after surgery in both groups and corresponding PODs

Group Designation	No. of Patients with fever	POD 0	POD 1	POD 2	POD 3	POD 4
TKA group	45	0	4(9%)	17(38%)	15(34%)	9(20%)
THA group	46	1(2%)	30(65%)	10(22%)	2(4%)	3(7%)

In the THA group of patients, 65% (*N* = 30) of patients had maximum body temperature on POD1, 22% (*N* = 10) on POD2, 4% (*N* = 2) on the POD3, and 7% (*N* = 3) on the POD4. 2% (*N* = 1) on POD1 (See Figs. 3, 4 and Table 3).

There is a statistically significant correlation between post-operative CRP levels and the development of post-operative fever in the TKA group (*P* < 0.05) (see Table 4). The higher the CRP level, the more likely you are to develop a fever after surgery. The similar finding was also reached by the THA group. Furthermore, we also discovered that fall in hemoglobin is related to fever (*P* < 0.05) (see Table 5).

**Table 4:** Comparison between perioperative febrile-related factors of the afebrile group and febrile group (TKA)

Factor	Afebrile group(*n* = 90)	Febrile group(*n* = 45)	t value	*p* value
Surgical time (min), mean ± SD	81.74 ± 18.33	82.88 ± 18.11	0.326	0.745
Postoperative drainage volume(ml), mean ± SD	190.76 ± 133.32	232.66 ± 186.48	1.362	0.177
Fall in hemoglobin (g/L), mean ± SD	16.59 ± 9.88	18.40 ± 12.28	0.892	0.374
Pre-operative albumin(g/L), mean ± SD	41.95 ± 3.89	40.81 ± 3.11	1.647	0.102
Post-operative CRP (mg/L), mean ± SD	13.81 ± 10.68	61.67 ± 45.18	5.839	< 0.001
Gender (male/female)	23/67	9/36	0.512	0.474
Hypertension (yes/no)	36/54	25/20	2.931	0.087
Diabetes (yes/no)	10/80	7/38	0.538	0.463
Transfusion (yes/no)	7/83	2/43	0.134	0.714
Urinary catheter (yes/no)	11/79	4/41	0.338	0.561
Anesthetic type(spinal/General)	30/60	9/36	2.596	0.107
Pre-operative albumin(g/L) (≥ 35 g/L, < 35 g/L)	85/5	42/3	< 0.001	1.000

**Table 5:** Comparison between perioperative febrile-related factors of the afebrile group and febrile group (THA)

Factor	Afebrile group(*n* = 92)	Febrile group(*n* = 46)	t value	*p* value
Surgical time (min), mean ± SD	80.39 ± 20.85	80.96 ± 20.54	0.151	0.880
Postoperative drainage volume(ml), mean ± SD	171.43 ± 123.30	174.48 ± 188.14	0.101	0.920
Fall in hemoglobin (g/L), mean ± SD	20.12 ± 10.29	25.95 ± 13.73	2.402	0.019
Pre-operative albumin(g/L), mean ± SD	41.51 ± 4.55	40.83 ± 4.62	0.825	0.411
Post-operative CRP(mg/L), mean ± SD	51.81 ± 38.98	94.31 ± 42.13	5.392	< 0.001
Gender (male/female)	38/54	25/21	2.103	0.147
Hypertension (yes/no)	29/63	16/30	0.148	0.700
Diabetes (yes/no)	7/85	6/40	0.520	0.471
Transfusion (yes/no)	4/88	3/43	0.109	0.891
Urinary catheter (yes/no)	14/88	10/36	1.498	0.221
Anesthetic type(spinal/General)	31/61	11/35	1.386	0.239
Pre-operative albumin(g/L) (≥ 35 g/L, < 35 g/L)	85/7	41/5	0.103	0.749

## Discussion

Fever is a fairly common symptom in the first few days after various types of surgery, including joint replacement surgery. At present, fever following joint replacement is thought to be caused by: ① non-infectious factors, such as inflammatory reactions generated by different cytokines in human body [9, 10]; ② Infectious factors such as incision infection, periprosthetic infection, urinary/ respiratory tract infection and so on [11]. Above all, the surgeon's primary concern is that a deep infection of the wound or prosthesis might occur. Because PJI is a catastrophic complication that might occur after total joint arthroplasty. However, our concern often leads to unnecessary diagnostic examinations such as chest radiographs, urine analysis, urine and blood cultures [3, 6, 12–14]. These examinations seldom create useful diagnostic value but will increase the financial burden of patients.

The quantity of publications focused on pyrexia following TJI is quite limited, with some different conclusions. Athanassious et al. [8] studied on 195 TKA patients and 146 THA patients, respectively. What they found was a 36% fever occurrence among patients. In addition, POD 1 was the day when a patient would most likely develop a fever, and POD 2 was the day when a maximum temperature would be measured on a patient body. They believed that fever was a normal phenomenon and should not cause a serious concern in the immediate postoperative period in most patients. It might be unnecessary to conduct urinalysis or urine culture in the evaluation of a fever in the immediate postoperative period. Chest x-ray would not become necessary unless the patient had suffered multiple fever attacks during his/her hospital stay.

Lu et al. [5] researched on 579 TKA patients and 383 THA patients. They found that fever occurred in 48.2% of patients (≥ 38℃), most commonly on POD1, and the maximum body temperature also most commonly appeared on POD1. Besides, they drew a conclusion that consecutive days of fever and fever ≥ 39℃ had something to do with infection in patients after TJA.

Kennedy et al. [15] investigated 90 TKA patients and discovered that 17% of patients experienced fever (≥ 39℃), most commonly on POD1. Their findings were: There were significant independent correlations between high pyrexia and both blood transfusion and fall in haematocrit; Pyrexia and the presence of an infective focus were irrelevant. And they concluded that pyrexia occurring in the first few days following TKR was a normal physiological process. A recent study investigated 168 TKA patients and reached a conclusion that the temperature rose most remarkably on postoperative days 1 and 2, but only 4 patients caught fever up to 39 ℃. This study also showed an negative correlation between hemoglobin loss and postoperative body temperature [16].

Ghosh et al. [7] conducted a research on 170 TKA patients to find that patients’ fever occurrence rate was 36.5% (≥ 38℃), and that there was no relation between pyrexia and infection, allogenic blood transfusion, haemoglobin loss, use of urinary catheter, rheumatoid arthritis, anesthetic type, or previous pyrexia following TKA. Pyrexia in the first 5 days after TKR was usually a normal physiological response and excessive concern about the presence of infection would be unnecessary.

Shaw and Chung [17] examined 100 patients who had undergone TKA and another 100 patients total hip replacement (THA). They found that the average maximum temperature peaked on the POD1 and that 19% had a fever of 39 °C or above at some point, while achieved no finding of correlation between blood loss or transfusion and pyrexia was found. Therefore their final conclusion was that post-operative fever after TJA was a normal physiological response and that a full work-up for fever alone was not justified.

This study shows that only 4% of patients experienced a fever after TJA (3% of patients experienced a fever after TKA and 6% of patients experienced a fever after THA). In contrast, the prevalence of fever in the current study is far lower than previous studies. We believe that low pyrexia rate may be related to the widely used tranexamic acid or other hemostatic drugs. Besides, the patients in this study were treated on PODs 2 to 4 with NSAIDs for pain inhibition. This may also be a potential cause for low fever rates.

In addition, this study reveals that the first fever after TKA was most common on POD2, and the first fever after THA was most common on POD1. The maximum body temperature was most common on POD2 in patients with fever after TKA, and POD1 in patients with fever after THA. For each group, the first fever and the maximum body temperature occurred on the same day, which is consistent with study reported by Tai et al. [16]. in patients after TKA. The timing of maximal body temperature and first fever is likely due to surgical trauma and secondary systemic inflammatory responses related, which decreases with time as reflected by body temperature reduce.

We found no significant correlation between the development of a fever and hypertension, diabetes, blood transfusion, use of urinary catheter, an aesthetic type, surgical time, postoperative drainage volume and pre-operative albumin. This is consistent with the conclusion of Andres et al. [10]. While there was a significant correlation between fall in hemoglobin and the development of a fever in patients after THA, but it did not occur in patients after TKA. This is consistent with the conclusion of Ghosh et al. [7, 16, 18] in patients after TKA. The difference may be related to the use of tourniquet, because many studies had indicated that tourniquet application could reduce surgical time, intraoperative blood loss and total blood loss. Therefore, when performing THA surgery, we need to carefully stop the bleeding and apply hemostatic drugs to reduce the loss of hemoglobin. In addition, when there is excessive blood loss during the operation or the blood test indicates that the hemoglobin loss is excessive, blood transfusion should be timely. The above measures may avoid the occurrence of postoperative fever. But when blood transfusion will reduce the occurrence of postoperative fever is still unclear.

Furthermore, we found that there was significant association between the development of a fever and post-operative CRP. Surgical stress, postoperative infection and tissue injury can increase the level of serum C-reactive protein [19]. The smaller the surgical trauma was, the lower the blood C-reactive protein level would be. There was a positive correlation between the increase of body temperature and the increase of C-reactive protein after joint replacement. C-reactive protein is an important marker of inflammatory response, which is synthesized by hepatocytes. Its response often precedes clinical symptoms, including fever [20]. Therefore, we can take corresponding measures to reduce the level of postoperative inflammatory response. Adding steroids to peri-operative drug protocols is effective in reducing postoperative CRP levels [21], which may potentially reduce the occurrence of postoperative fever.

We followed these 2482 patients for at least 1 year and found that a total of 3 patients developed periprosthetic infections (all in the afebrile group). Due to the low incidence of PJI, we were unable to accurately determine the relationship between postoperative fever after TJA and PJI. However, our conclusions suggest that fever after TJA is a normal physiological response and does not increase the chance of PJI. This is also supported by several previous studies [7, 8, 15, 17]. When patients with postoperative fever have no clinical manifestations of incision infection, urinary tract infection, and respiratory tract infection, we do not need to worry too much about the occurrence of PJI, nor do we need to conduct a comprehensive examination of fever. Only symptomatic treatment of fever is sufficient.

### Limitations of this study

There are some limitations to our study. First of all, this is a retrospective analysis. Second, all the patients in this study were treated with non-steroidal anti-inflammatory drugs for analgesia, which might have alleviated the fever response of the patients. Third, all patients received antibiotics on POD0 ~ 2, which might also have reduced the febrile response.

## Conclusion

In conclusion, results of this retrospective analysis are different from the previous ones. We find that postoperative fever in patients after TJA is uncommon, and that first fever and the maximum body temperature most commonly occur on POD2 in patient after TKA, while POD1 in patient after THA. There is positive correlation between fever and C-reactive protein, the higher the C-reactive protein level is, the higher the incidence of postoperative fever will be. There is a significant association between fall in hemoglobin and the development of a fever in patients after THA. Therefore, there are a lot of ways to lower the incidence of postoperative fever, such as reducing C-reactive protein as much as possible and applying hemostatic drugs to reduce postoperative HGB loss, so as to avoid the waste of medical resources, and reduce the pain as well as economic pressure on patients.

POF after TJA is a normal physiological response and does not increase the chance of PJI. When incision infection, urinary tract and respiratory tract infection are not considered, only symptomatic treatment is required.

## Data Availability

The datasets generated and/or analyzed during the current study are not publicly available due but are available from the corresponding author on reasonable request.

## Abbreviations

POF: Postoperative fever
TJA: Total joint arthroplasty
TKA: Total knee arthroplasty
THA: Total hip arthroplasty
POD: Postoperative Day
PJI: Periprosthetic joint infection
CRP: C-reactive protein
ESR: Erythrocyte sedimentation rate
PCT: Procalcitonin
DVT: Deep venous thrombosis
